# Genome-wide characterization, evolution and expression profiling of UDP-glycosyltransferase family in pomelo (*Citrus grandis*) fruit

**DOI:** 10.1186/s12870-020-02655-2

**Published:** 2020-10-07

**Authors:** Boping Wu, Xiaohong Liu, Kai Xu, Bo Zhang

**Affiliations:** 1 The Key Laboratory for Quality Improvement of Agricultural Products of Zhejiang Province, College of Agriculture and Food Science, Zhejiang Agriculture and Forestry University, Hangzhou, 311300 China; 2 Zhejiang Provincial Key Laboratory of Horticultural Plant Integrative Biology / Laboratory of Fruit Quality Biology, Zhejiang University, Hangzhou, 310058 China

**Keywords:** UDP-glycosyltransferase, Expression analysis, Pomelo, Evolution, Glycosylation

## Abstract

**Background:**

Pomelo is one of the three major species of citrus. The fruit accumulates a variety of abundant secondary metabolites that affect the flavor. UDP-glycosyltransferases (UGTs) are involved in the glycosylation of secondary metabolites.

**Results:**

In the present study, we performed a genome-wide analysis of pomelo *UGT* family, a total of 145 UGTs was identified based on the conserved plant secondary product glycosyltransferase (PSPG) motif. These *UGT* genes were clustered into 16 major groups through phylogenetic analysis of these genes with other plant UGTs (A-P). Pomelo UGTs were distributed unevenly among the chromosomes. At least 10 intron insertion events were observed in these *UGT* genome sequences, and I-5 was identified to be the highest conserved one. The expression profile analysis of pomelo *UGT* genes in different fruit tissues during development and ripening was carried out by RNA-seq.

**Conclusions:**

We identified 145 UGTs in pomelo fruit through transcriptome data and citrus genome database. Our research provides available information on UGTs studies in pomelo, and provides an important research foundation for screening and identification of functional *UGT* genes.

## Background

Plants produce a variety of secondary metabolites that are involved in important mechanisms at different developmental stages. As an important modification reaction, glycosylation is usually the last step in the biosynthesis of secondary metabolites in plants. In coordination with methylation, hydroxylation, and acylation, glycosylation contributes to the diversity and complexity of secondary metabolites in plants [1]. Glycosylation is the main mechanism for stabilizing and enhancing the solubility of metabolites in plants, thereby regulating plant signaling pathways and intracellular homeostasis [2, 3].

Glycosyltransferase catalyzes glycosylation [4]. In 2020, a total of 110 glycosyltransferase (GT) super families were included in the CAZy database (http://www.cazy.org), of which the GT1 family had the largest number of gene members and is mainly in plants. The GT1 family is commonly referred to as UDP-glycosyltransferase (UGT), because primarily it catalyzes the transfer of UDP-sugars to specific receptors, including plant hormones, secondary metabolites, and xenobiotics [5]. Plant UGT has a conserved sequence of 44 amino acids at the C-terminus, named the plant secondary product glycosyltransferase box (PSPG box), which is responsible for the binding of glycosyl groups [5]. The N-terminus, which varies considerably in sequence, is responsible for the recognition of different receptor molecules [1].

UGTs involved in plant secondary metabolism often show a wide range of substrate specificities. Several *UGT* genes were functionally characterized to glycosylated terpenoids, phenylpropanoids, and flavonoids in fruits. The phloretin-specific glycosyltransferase UGT88F1 was identified in apple [6]. VvGT5 and VvGT6 from grapevine [7], FaGT6 and FaGT7 from strawberry [8], were identified as being involved in the glycosylation of flavonoids. AdGT4 was identified as glycosylating terpene alcohols in kiwifruit [9], while F3GT1 was involved in the biosynthesis of kiwifruit anthocyanins [10]. In peach fruit, PpUGT85A2 was identified as glycosylating terpenoids and phenylpropanoids [11] and PpUGT78A1 and PpUGT78A2 as being responsible for glycosylation of anthocyanins [12]. In citrus, some UGT members that catalyze flavonoid glycosylation have been reported, including Cm1_2RhaT [13] from pomelo, CsUGT76F1 [14] and Cs1,6RhaT [15, 16] from sweet orange, and three other UGTs were considered to be putative terpenoid glycosyltransferases [17].

To date, complete and nearly complete genome sequencing programs have been available for many plant species, providing the basis for the genome-wide research of the *UGT* gene family. It has been found that *UGT* exists in plants as multigene families and has expanded in many plant species. More than 100 *UGT* members have been identified in *Arabidopsis*, and even more *UGT* family members have been found in other plant species. For example, 147, 179, and 180 *UGTs* have been identified in *Zea mays* [18], *Triticum aestivum* [19], and *Oryza sativa* [5], respectively. In addition, for fruits, genome-wide analysis of *UGT* gene families has also reported, including in *Malus* x *domestica* [5], *Vitis vinifera* [5], and *Prunus persica* [20]. However, information on UGT in other fruits is still limited.

Pomelo is one of the most important economic citrus species and the fruit contain special flavors and are rich in flavonoids which are good for human health. In this study, 145 *UGT* gene members were identified in pomelo based on genome database analysis. Phylogenetic analysis, chromosome localization and gene structure of Extron-intron analysis were carried out. Expression patterns of pomelo *UGT* genes in various fruit tissues during development and ripening were analyzed by RNA-seq. Genome-wide analysis of *UGT* gene family provides insights for future screening and functional identification of pomelo UGTs.

## Results

### Identification of pomelo UGTs

A total of 145 UGTs was identified in pomelo fruit as containing a consensus sequence (PSPG box) at the C-terminus of the protein. These *UGT* genes encoded predicted proteins ranging from 144 to 680 amino acids (average 459 amino acids) (Table S1). The molecular weight from 16.39 to 76.87 kDa (average Mw = 51.26 kDa) and the isoelectric point (pI) ranged from 4.82 to 9.18 (average pI = 5.81). Subcellular localization of these genes indicated that 88 UGT members (61% of UGTs) were probably in the cytoplasm, and 35 (24%) and 17 (12%) UGTs were most probably in the plasma membrane and chloroplast, respectively. Only one UGT (Cg7g000340) was predicted to be located in the mitochondria, two (Cg6g025740 and Cg8g023190) in the nucleus and two (Cg3g014800 and Cg3g014820) extracellular (Fig. S1, Table S2).

### Phylogenetic analysis of pomelo UGTs

**Fig. 1:**
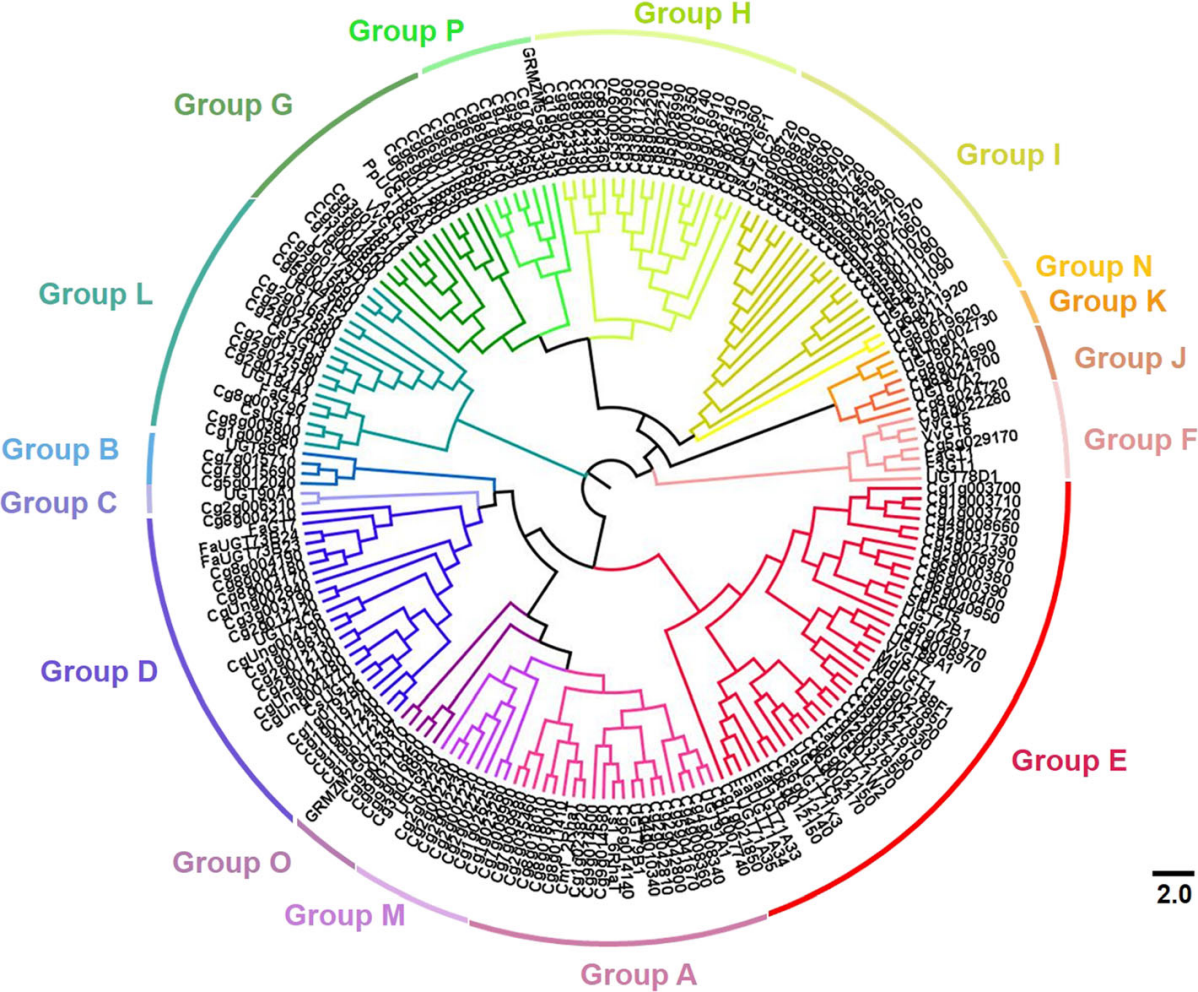
Phylogenetic analysis of *UGT* family genes from pomelo. The tree was constructed using the neighbor-joining method by aligning the amino acid sequences of pomelo UGTs with several plant functional UGTs from Arabidopsis, citrus, maize, tomato, grapevine, peach, apple, kiwifruit and strawberry. The phylogenetic tree was visualized using the FigTree v1.4.2 program. Each of the 16 groups (A-P) is indicated in a specific color

In order to explore the evolutionary relationships of plant UGT families, the phylogenetic tree was constructed based on the pomelo and other plant UGT protein sequences, including *Arabidopsis*, citrus, maize, tomato, grapevine, peach, apple, kiwifruit and strawberry (Fig. 1). All UGT members were divided into 16 phylogenetic groups, including 14 conservative groups (A-N) identified in *Arabidopsis* [2], and two newly identified groups O and P found in other plants, such as grapevine [5]. Cm1_2RhaT (100% amino acid sequence identity to Cg1g023820) from pomelo and Cs1,6RhaT from sweet orange that were identified as flavonoid 7-*O*-UGTs [13, 15, 16], were clustered in group A. CsUGT76F1, located in group H, was identified as being involved in the biosynthesis of flavonoid 7-*O*-glucosides and 7-*O*-rhamnosides in sweet orange [14]. *Arabidopsis* UGT73C6 (flavonol-3-*O*-rhamnoside-7-*O*-glucosyltransferase) [21] and strawberry FaGT7 (flavonol-3-*O*-glucosyltransferase) [8] were located in group D. Other UGTs responsible for flavonol-3-*O*-glycosylation were located in group F, including UGT78D1 from *Arabidopsis* [22], and VvGT5 and VvGT6 from grapevine [7].

Three putative terpenoid UGTs were isolated in ‘Valencia’ sweet orange, CsUGT1 and CsUGT3 were clustered in group L, while CsUGT2 was clustered in group D [17]. Several UGT73 (belonging to group D) and UGT71 family members (belonging to group E) were functional in the biosynthesis of anthocyanins and the glycosylation of volatile metabolites, including terpenoids [23, 24]. Some other UGTs in group G also have been functionally characterized as participating in terpenoid glycosylation and affecting fruit flavor, such as kiwifruit AdGT4 [9], grapevine VvGT14 [25], and peach PpUGT85A2 [11].

### Distribution of plant UGTs in phylogenetic groups

**Fig. 2:**
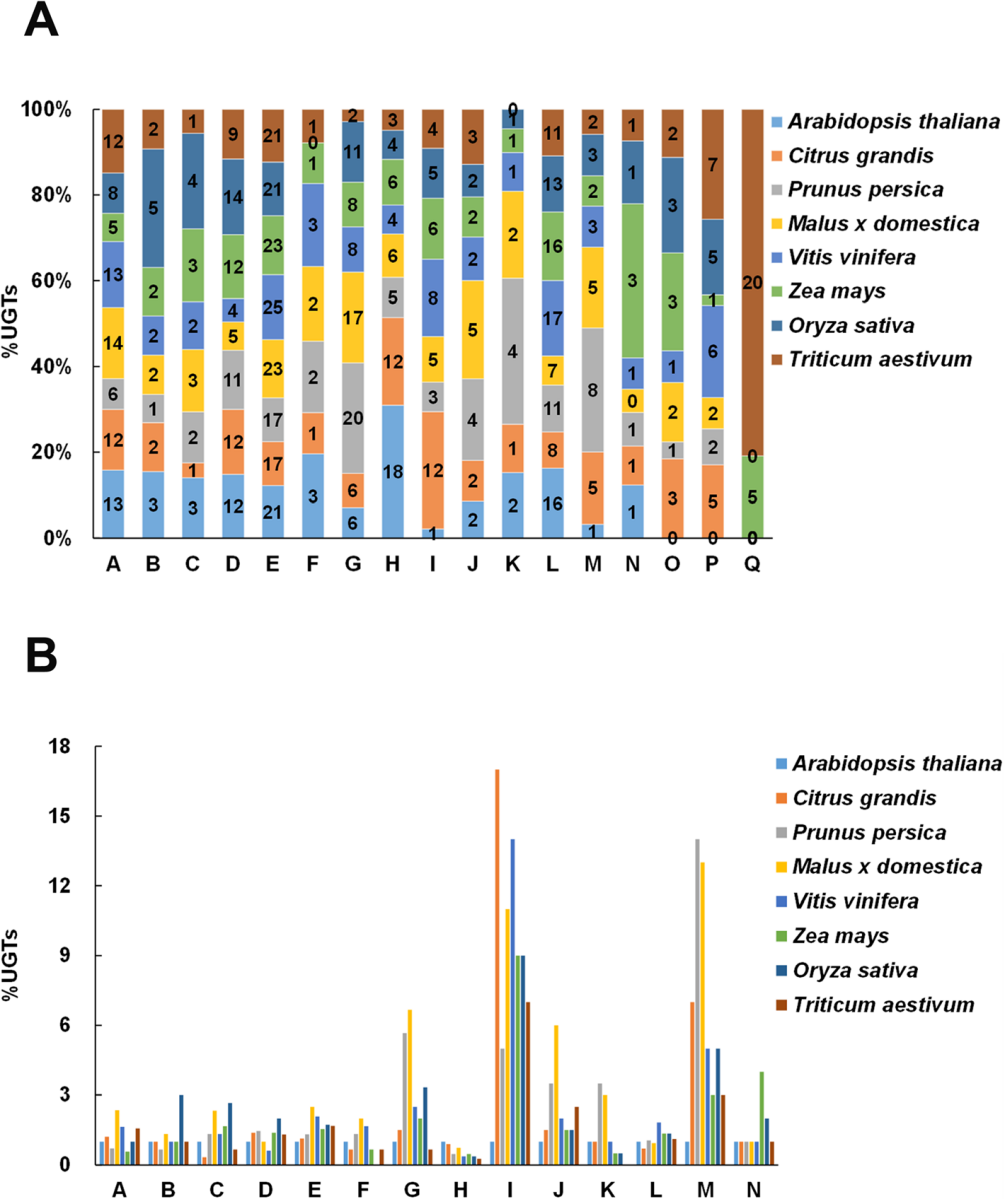
Expansion of the plant UGTs in several plant species. **a** The percentage of plant UGTs in the different phylogenetic groups. **b** Comparison of the fold increase of plant UGTs in each group with reference to *Arabidopsis thaliana*. Genes from different species are indicated by specific colors

The evolutionary pattern of the plant *UGT* gene family was analyzed by comparing the distribution of UGTs in the different phylogenetic groups (Table 1). During the evolution of higher plants, the five phylogenetic groups A, D, E, G, and L appeared to expand more than others, although the number of genes found in these groups varies widely among species. In pomelo, six phylogenetic groups, A, D, E, H, I, and L expanded more than the other groups. There were only 9 pomelo UGTs in group G, more than in *Arabidopsis* (6 UGTs), but much fewer than in other plants, especially peach and apple, which had up to 34 and 40 UGTs respectively in group G. The number of pomelo UGTs in the group I accounted for 12% of the total pomelo UGTs, much higher than in other plants (Fig. 2a). The proportion of pomelo UGTs in group H (about 12% of the total pomelo UGTs) was much higher (1.5 ~ 12 fold) than in other fruits such as peach (*Prunus persica*), apple (*Malus* x *domestica*) and grapevine (*Vitis vinifera*) (Fig. 2a).

**Table 1:** Number of the plant UGTs in the different phylogenetic groups

UGT group	*Arabidopsis thaliana*^a^	*Citrus grandis*	*Prunus persica*^*b*^	*Malus* x *domestica*^a^	*Vitis vinifera*^a^	*Zea mays*^c^	*Oryza sativa*^a^	*Triticum aestivum*^*d*^	Total
A	14	17	10	33	23	8	14	22	141
B	3	3	2	4	3	3	9	3	30
C	3	1	4	7	4	5	8	2	34
D	13	18	19	13	8	18	26	17	132
E	22	25	29	55	46	34	38	37	286
F	3	2	4	6	5	2	–	2	24
G	6	9	34	40	15	12	20	4	140
H	19	17	9	14	7	9	7	5	87
I	1	17	5	11	14	9	9	7	73
J	2	3	7	12	4	3	3	5	39
K	2	2	7	6	2	1	1	–	21
L	17	12	18	16	31	23	23	19	159
M	1	7	14	13	5	3	5	3	51
N	1	1	1	1	1	4	2	1	12
O	–	4	1	5	2	5	6	3	26
P	–	7	4	5	11	1	9	13	50
Q	–	–	–	–	–	7	–	36	43
Total	107	145	168	241	181	147	180	179	1348

^a^Data from *Caputi* et al. *(2012)*; ^b^Data from *Wu* et al. *(2017)*; ^c^Data from *Li* et al. *(2014)*; ^d^Data from *Liu* et al. *(2019)*

It was worth noting that the number of plant UGTs in group I and group M was significantly increased by comparing the number of plant UGTs in each phylogenetic group with those reported in *Arabidopsis* (Fig. 2b). In *Arabidopsis*, there was only one UGT member in group I, while other plants contained 5–17 members, and the number of UGTs in pomelo was the highest (Table 1; Fig. 2b). In group M, the number of UGTs ranged from one in *Arabidopsis* to 14 in peach, and there was a 7-fold difference between *Arabidopsis* and pomelo. In addition, the number of pomelo UGTs in some groups was reduced relative to *Arabidopsis*, including group C, F, H and L.

### Chromosomal location of *UGT* genes in pomelo

**Fig. 3:**
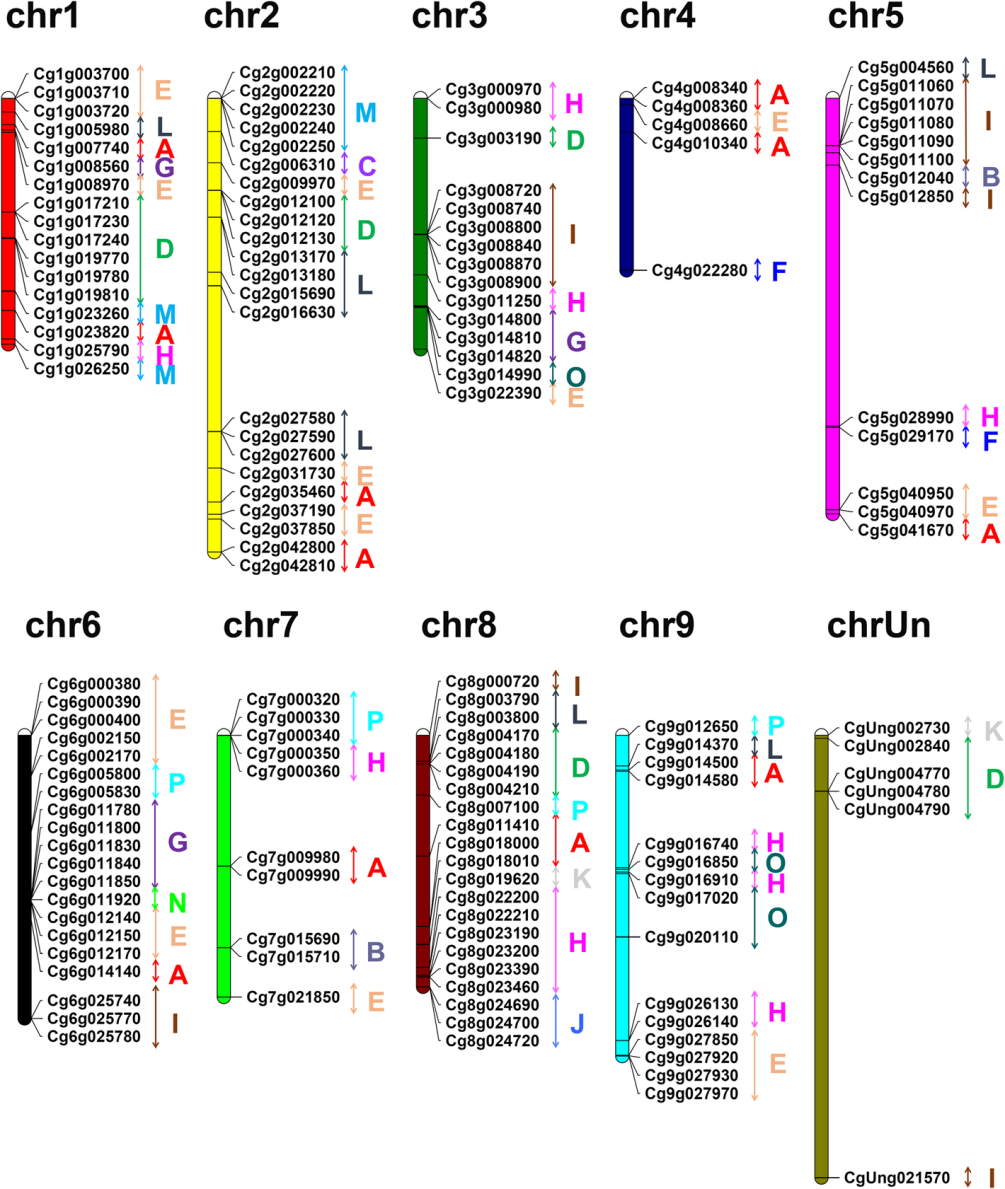
Chromosome distribution of pomelo UGTs. The pomelo UGTs were distributed among 9 chromosomes (chr1-chr9) and one unknown chromosome (chrUn). The different colored bars represent a chromosome, and chromosome numbers are displayed at the top of each chromosome. Different color letters represent different phylogenetic groups

To summarize the genomic distribution of pomelo *UGT* genes, the chromosomal location of *UGT* genes was investigated based on the genome annotation information retrieved from the pomelo genomic databases (Fig. 3; Table S3). A total of 139 *UGT* genes were unevenly distributed on the pomelo genome of 9 chromosomes, the remaining 6 *UGT* genes were localized on the unknown chromosome (chrUn), including *CgUng002730* of group K, *CgUng021570* of group I, and four *UGT* genes of group D. In the pomelo genome, chromosome 2 contained the most *UGT* genes (23 members), followed by 21 members located on chromosome 8, and 20 members on chromosome 6. Only five members were distributed on chromosome 4, which contained the least number of *UGT* genes.

Since pomelo UGTs could be divided into 16 groups, the localization of these groups on the chromosomes was observed (Fig. 3). The *UGT* genes of group E with the most members (25 genes) were randomly distributed across eight chromosomes (chromosome 1–7 and 9). For group I, chromosome 3 and 5 each contained six *UGT* genes, chromosome 6 had three *UGT* genes, and the remaining two members were located on chromosome 8 and unknown chromosome. Group M contained 7 *UGT* genes, five of which were located on chromosome 2 and two were located on chromosome 1.

### Structural analysis of *UGT* genes in pomelo

To better explore the relationships among the structure and function of pomelo *UGT* genes, and further clarify the evolutionary relationships within the *UGT* gene family, the exon/intron structure was analyzed. Among the 145 pomelo *UGT* genes, 70 *UGTs* (48%) had no introns, 63 *UGTs* (43%) contained one intron, whereas the remaining eight *UGTs* contained two introns, and three *UGTs* contained three introns, only one *UGT* contained eight introns (Table 2; Fig. S2). For *UGT* groups, group E contained the largest number of genes with losing introns (22 members), followed by 15 in group D and 13 in group A. All members of group B, C and O had no introns. Most of the *UGTs* in group H and I contained one intron with 14 UGTs out of all 17 members (82%) in each group.

**Table 2:** Number of pomelo UGT genes in each group according to number of introns

Group	No. of Introns					Total
	0	1	2	3	8	
A	13	4	0	0	0	17
B	3	0	0	0	0	3
C	1	0	0	0	0	1
D	15	1	1	0	1	18
E	22	3	0	0	0	25
F	1	1	0	0	0	2
G	0	8	1	0	0	9
H	0	14	2	1	0	17
I	0	14	1	2	0	17
J	0	2	1	0	0	3
K	0	2	0	0	0	2
L	4	6	2	0	0	12
M	6	1	0	0	0	7
N	0	1	0	0	0	1
O	4	0	0	0	0	4
P	1	6	0	0	0	7
Total	70	63	8	3	1	145

**Fig. 4:**
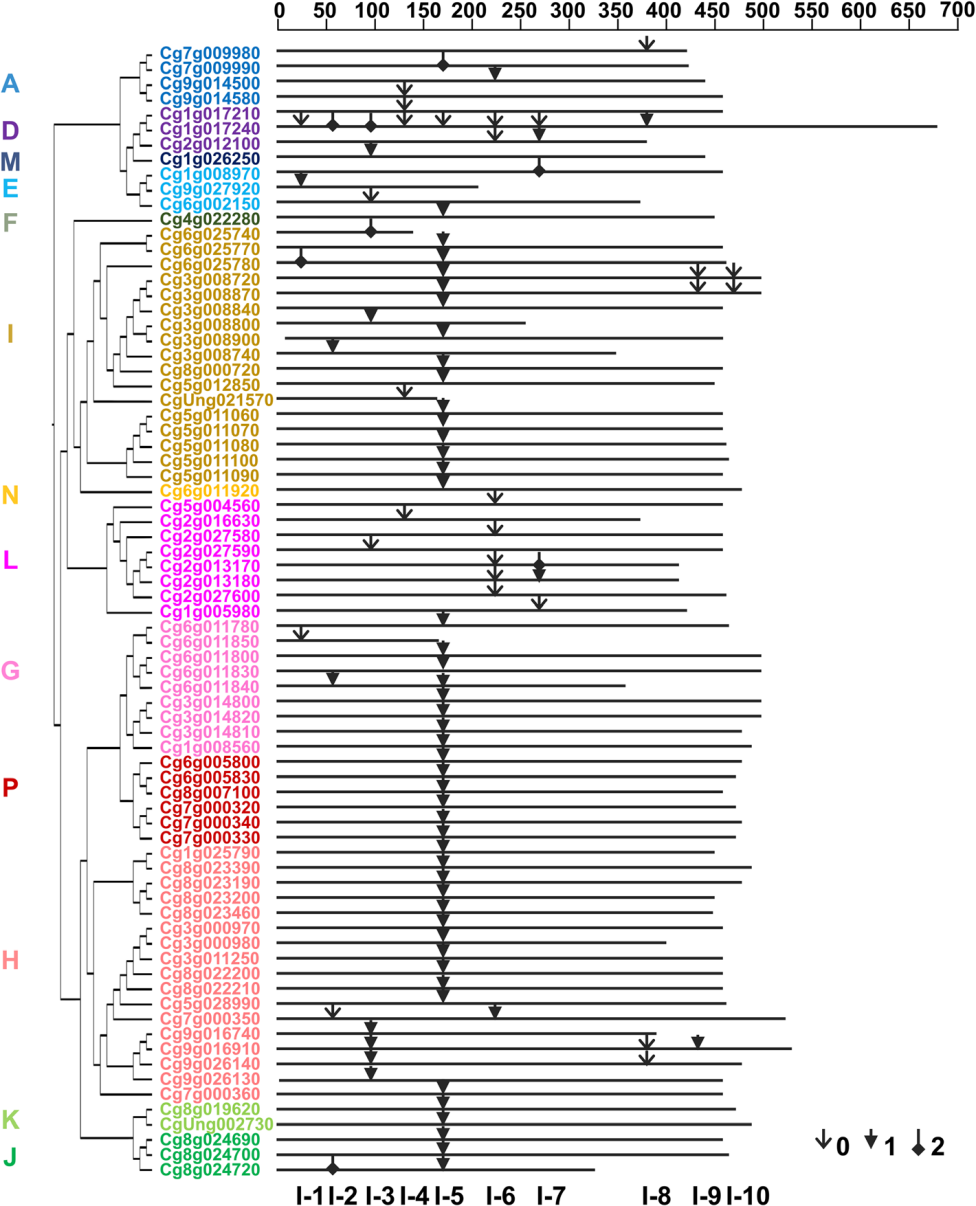
Distribution of introns among 75 pomelo *UGT* genes. The introns were mapped and numbered according to the alignment of the encoding sequences for the *UGT* genes. The position of introns inserted into each gene is represented by the scale on the top of the map. The gray lines indicate the length of their amino acid sequences. Arrows indicate the position of intron insertion in each gene. Intron phases are represented by different shapes of arrows for 0, 1, and 2, respectively. Phylogenetic relationship on the left indicate the classification of pomelo *UGT* genes

After searching for all of the 75 intron-containing sequences and mapping the introns to the amino acid sequences, 10 independent intron insertion events were observed in the pomelo *UGT* gene family members (Fig. 4). Based on the positions in the protein sequences, these insertion events were numbered sequentially from I-1 to I-10. Intron 5 (I-5) was indicated to be a highest conserved intron, which was the most widespread intron of *UGTs*, containing 48 *UGT* members (64% of the intron containing *UGTs*), except for group E, L and M. All members of group F, K, J, N and P, and most members of group G, H and I contained the intron 5. A total of eight of the nine *UGTs* in group G contained intron 5, 13 of 17 in group I, and 12 of 17 in group H. Intron 6 was mainly observed in group L.

Most of the total 96 introns identified in the *UGT* gene structures of pomelo were in phase 1, accounting for 64% (61 introns), followed by 28% (27 introns) in phase 0, and only 8% (8 introns) in phase 2 (Fig. 4). For the highly conserved intron 5, each had one intron in phase 0 and 2, while phase 1 accounted for 96% of all introns. All members of intron 4 and 7 out of 9 *UGTs* with intron 6 were in phase 0. These findings indicated that most of the high conserved introns were ancient elements and their phases remained stable during evolution.

### Expression profiles in different fruit tissues during development and ripening

**Fig. 5:**
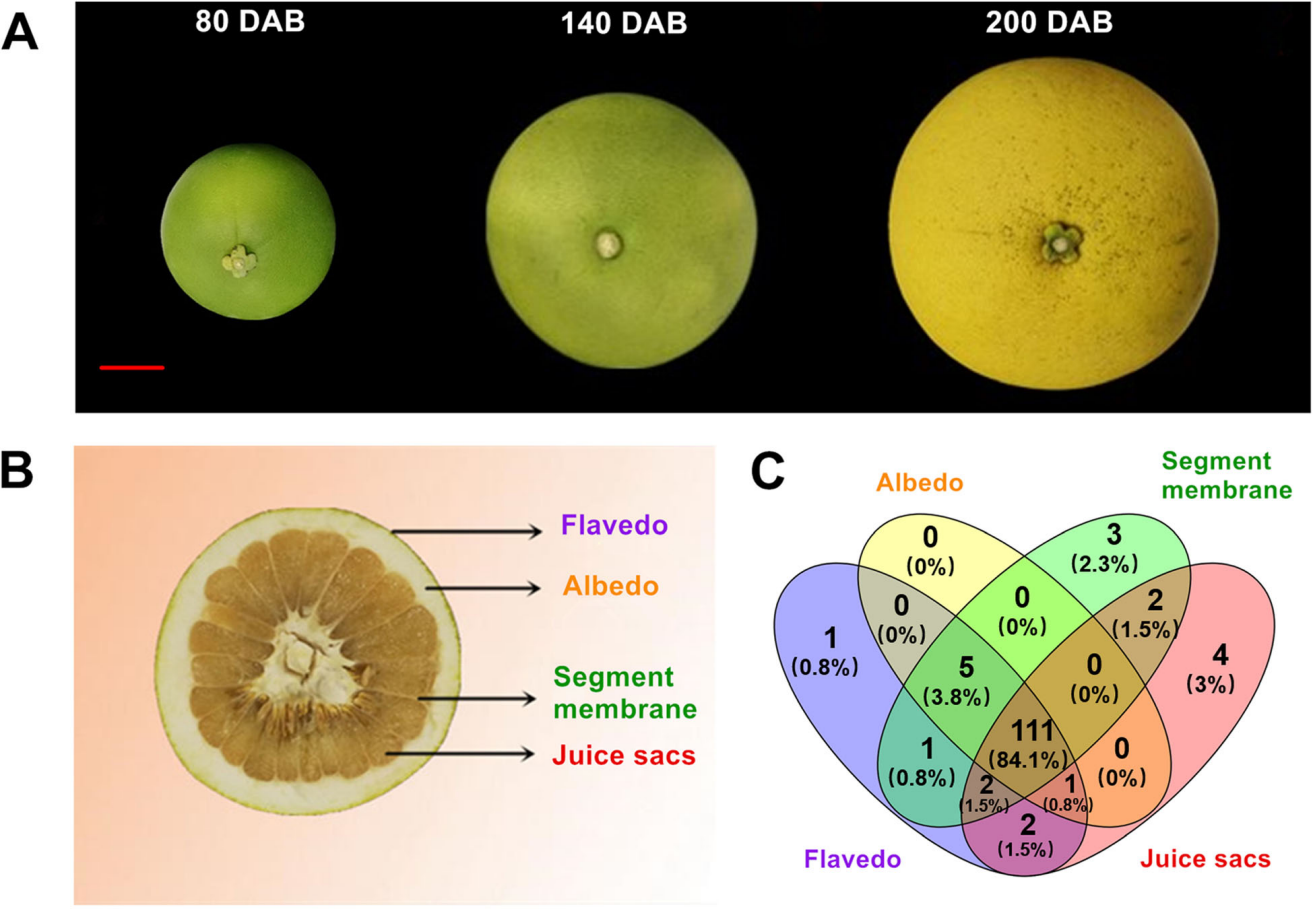
Ripening pomelo fruit and Venn diagram of pomelo *UGT* genes. **a** Photo of pomelo fruit during three developmental stages: green stage at 80 days after full blossom (DAB), color breaking stage at 140 DAB, and mature stage at 200 DAB. **b** Four tissues of pomelo fruit: flavedo (the outer skin), albedo (the inner pith), segment membrane and juice sacs. **c** Venn diagram of *UGT* genes in pomelo fruit. The distribution of 145 *UGT* genes in the four tissues of pomelo fruit

**Fig. 6:**
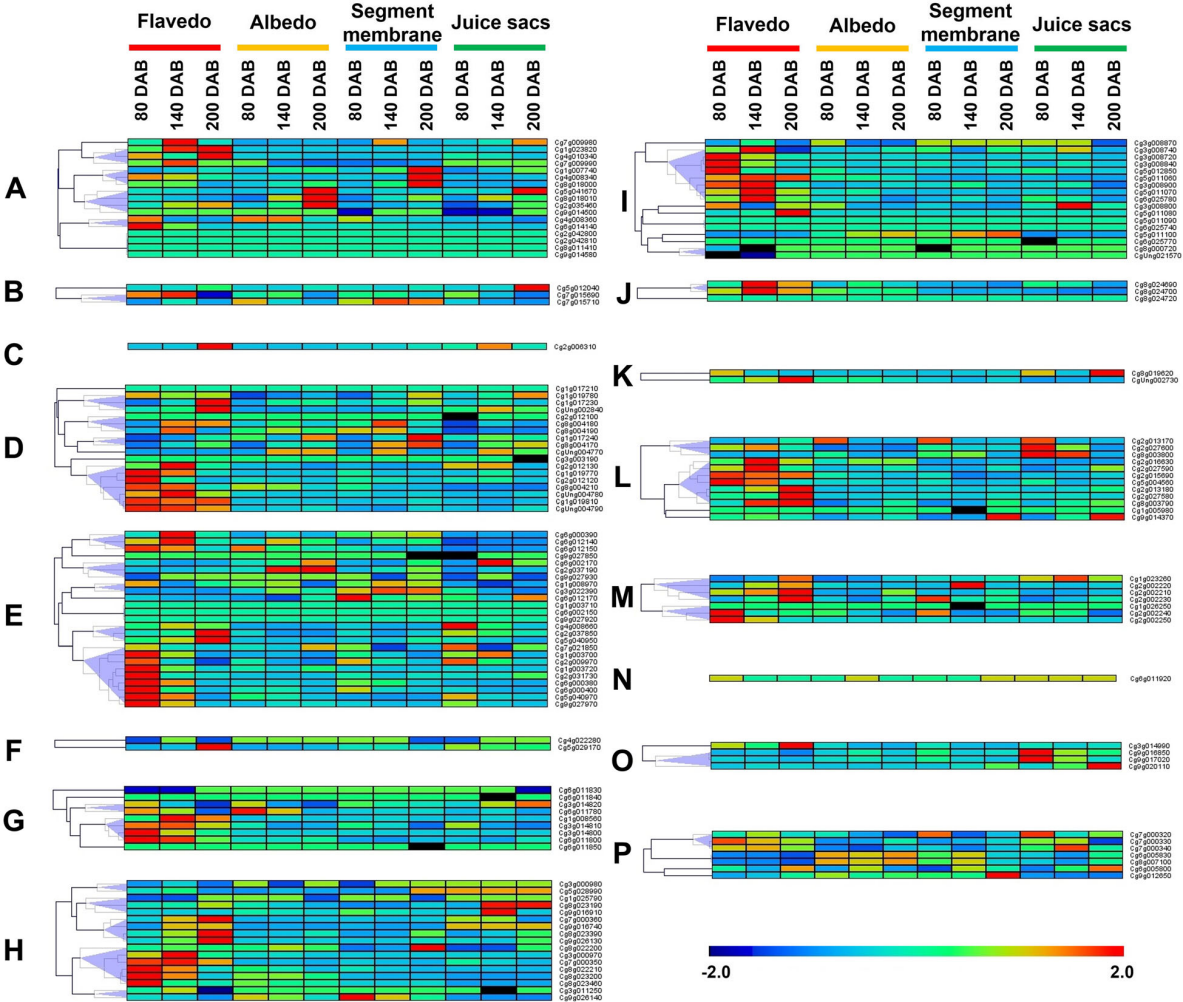
Transcript levels of *UGT* genes in pomelo fruit during development and ripening. The pomelo *UGT* genes expression profiles of different fruit tissues were studied during three developmental stages: green stage at 80 days after full blossom (DAB), color breaking stage at 140 DAB, and mature stage at 200 DAB. The heat map was calculated by hierarchical clustering of *UGT* genes. The transcript abundance of *UGT* genes is represented by the color scale (representing − 2 to 2). Different letters indicate different phylogenetic groups of pomelo *UGT* genes

To detect the expression profiles of 145 pomelo *UGT* genes, transcript abundances of *UGTs* in different fruit tissues during development and ripening were analyzed using RNA-seq data (Fig. 5; Fig. 6). The four different tissues of pomelo fruit were flavedo, albedo, segment membrane (SM) and juice sacs (JS) (Fig. 5a, b). A total of 111 *UGT* genes (accounting for 84.1% of total pomelo *UGTs*) were expressed in all four fruit tissues. Additionally, 4 *UGT* genes (accounting for 3%), three *UGT* genes (2.3%), and one *UGT* gene (0.8%) were specifically expressed in JS, SM, and flavedo, respectively, but no genes were specifically expressed in albedo (Fig. 5c).

For pomelo fruits at different developmental stages, nearly half (71 members) of the *UGT* genes showed the highest level of transcript in the flavedo (Fig. 6). Among them, 13 *UGTs* (52%) belonged to group E, 10 *UGTs* (56% of members in group D) belonged to group D, 8 *UGTs* (47%) in group H, 9 *UGTs* (53%) in group I, and 7 *UGTs* (58%) in group L. The expression levels of 29 members in pomelo were highest at green stage (80 DAB), followed by 23 at color break stage (140 DAB), and 19 at mature stage (200 DAB). *Cg1g023820* in group A had 100% identity with the amino acid sequence of *Cm1_2RhaT* from pomelo, which was identified to be a flavonoid 7-*O*-UGT [13, 15, 16], showed the highest transcript levels at color break stage (140 DAB) and mature stage (200 DAB) in flavedo of pomelo fruit. This finding was consistent with previous research. Considering the largest number of pomelo *UGT* genes in group E, the expression patterns in different fruit tissues during development and ripening were further analyzed (Fig. 6). A total of nine *UGT* genes showed the highest abundance of transcript at the green stage (80 DAB) of flavedo, two genes at the color break stage of flavedo, and two at the mature stage. Only one *UGT* gene, *Cg2g037190* expressed highest at the color break stage and mature stage of albedo, while the two genes *Cg3g022390* and *Cg6g012170* had the highest expression level in the segment membrane (SM) of pomelo fruit, and three genes predominantly expressed in the juice sacs (JS).

## Discussion

Plant glycosyltransferases belong to a large and functionally diverse family of enzymes characterized as glycosyltransferase family 1, also named UDP-glycosyltransferase (UGT) [1]. Plant UGTs catalyze a wide range of substrates for glycosylation reactions, including plant hormones, plant secondary metabolites and biological homogeneous/heterologous substances (such as cyanogenic glycosides and herbicides). Plant secondary metabolites are generally divided into three categories: phenols, terpenes, and nitrogen-containing organic compounds. Some of these compounds are chemically modified, and glycosylation is one of the important modification reactions. Several *UGT* genes have been functionally characterized in plants, such as *Arabidopsis thaliana* [5], *Zea mays* [18], *Triticum aestivum* [26], *Brassica rapa* [27] and *Prunus persica* [20]. In citrus, only three UGTs have been functionally identified, Cm1_2RhaT from pomelo, Cs1,6RhaT and CsUGT76F1 from sweet orange associated with flavonoid glycosylation [13–16], and three putative terpenoid UGTs, CsUGT1, CsUGT2 and CsUGT3 [17]. However, no large-scale analysis was found in citrus.

In this study, a total of 145 *UGT* genes was identified in pomelo fruit, accounting for about 0.5% of the pomelo gene product, which is lower than 0.6% of peaches [20]. The pomelo *UGT* genes were clustered into 16 groups, including 14 highly conserved groups (A-N) and two newly discovered O and P groups (Table 1). A new group Q was found only in maize and wheat, suggesting that this group may exist only in monocotyledons and play an important role in the *UGT* gene evolution of monocotyledons. In contrast, group K had only one *UGT* member in maize and rice, and no member in wheat, implying that group K may expand members in dicotyledons. Only one member was identified in the pomelo group N, which is the same as in dicotyledons such as *Arabidopsis*, apple and peach. This finding provides additional evidence for previous speculation that the group N was mainly amplified in monocotyledons [18, 20]. In addition, group E contained the most *UGT* genes, accounting for 17% of all *UGT* genes in pomelo. Furthermore, the *UGT* genes of group E accounted for the highest proportion in the eight species compared in this study, indicating that group E has been expanded in various plant species. In addition, many plant *UGT* gene members belonging to group E have been functionally identified, including the glycosylation of small molecule volatile compounds, and synthesis of flavonoid glycosides, phlorizin, and anthocyanins [6, 23, 24], which indicates that group E made an important contribution to the glycosylation of plant secondary metabolites.

Introns are an important part of genes, although they do not participate in the encoding of proteins, the intron gain or loss events and the insertion position of introns relative to protein sequences are generally considered as the key clues to understand the evolution or diversification of gene family [28]. Intron mapping of 145 pomelo *UGTs* revealed that 48% *UGT* members lacked introns, which is more than the number (43%) in peach [20], while less than the number (58%) in *Arabidopsis* [5]. A total of ten intron insertion sites was identified in the pomelo *UGT* genes, the same with those found in peach [20]. Among these introns, intron 5 (I-5) was considered to be the most widely distributed intron, except for groups E, L and M, and the two groups B and C without introns. For the peach 145 *UGTs* [20], wheat 179 *UGTs* [26] and maize 147 *UGTs* [18], intron 5 was also found to be the oldest intron. For the remaining introns, some were predominantly observed in certain phylogenetic groups, such as intron 6 was mainly present in group L, which is similar to the observation in peach [20]. Interestingly, a large quantity of intron 5 were in phase 1, while many of intron 6 were in phase 0, and the number of phase 0 and 1 introns was greater than the number of phase 2 introns. This finding was consistent with previous research, showing that conserved introns were ancient elements and intron phases were generally conservative during evolution, and can remain constant for many years, because any deletion and insertion of small DNA fragments that cause a phase change may lead to changes in gene function, and be eliminated by natural selection [29].

In previous studies, three *UGT* genes, *Cm1_2RhaT* from pomelo (*Citrus maxima*), *CsUGT76F1* and *Cs1,6RhaT* from sweet orange (*Citrus sinensis*) were functionally characterized to be involved in the biosynthesis of flavonoid 7-*O*-glucosides [15, 16]. Cm1_2RhaT showed 100% amino acid sequence identity to Cg1g023820 found in our study, CsUGT76F1 had 98.69% amino acid sequence identity with Cg7g000360, Cs1,6RhaT and Cg6g014140 had 81.26% identity. It was worth noting that Cg1g023820, Cg7g000360 and Cg6g014140 were specifically expressed in the flavedo of fruit, which is consistent with the high accumulation of glycosylated flavonoids in flavedo [30, 31]. Additionally, many (~ 50%) of the pomelo *UGT* genes showed the highest level of transcript in flavedo, indicating that *UGTs* play an important role in the biosynthesis of glycosylated secondary metabolites.

## Conclusions

This study provided useful insights into the evolution, distribution, gene structure, and expression profiling of pomelo UDP-glycosyltransferase. A total of 145 UGTs was identified in pomelo fruit. These genes were distributed unevenly among the 9 chromosomes, and clustered into 16 phylogenetic groups (A-P). Ten intron positions were observed in pomelo *UGT* genes, which indicated that they played an important role in the evolution and divergence of *UGT* gene family. The tissue-specific expression in four different fruit tissues during development and ripening was demonstrated by RNA-seq analysis, suggesting essential roles of UGTs in pomelo. This research would facilitate the screening of candidate genes and further characterization of their substrate specificity and biological function.

## Materials and methods

### Plant materials

Yuhuan (*C. grandis* (L.) Osbeck ‘Yuhuan’) pomelo fruits were harvested from the KEHAO Fruit Professional Cooperative in Yuhuan County, Zhejiang Province, and the sampling permissions were obtained. Fruit were harvested at three developmental stages, including green stage, 80 days after full blossom (DAB), color breaking stage at 140 DAB, and mature stage at 200 DAB. At each sampling time, the fruit was divided into four different parts: flavedo (the outer skin), albedo (the inner pith), segment membrane and juice sacs. Three biological replicates, each with five fruits were harvested, frozen in liquid nitrogen, and stored at -80^o^ C until further analysis.

### Identification of *UGT* genes

The pomelo UGTs were identified by using ‘UDP-glycosyltransferase’ as a query to screen genes in functional annotation results from transcriptome data. Further confirmation of UGTs was based on the 44-amino acid conserved motif of the plant secondary product glycosyltransferase box (PSPG box). The pomelo *UGT* gene sequences and the information of *UGTs*, including chromosome location, exon-intron structure and description were obtained from the citrus genome database (http://citrus.hzau.edu.cn/orange/). The length, molecular weight (Mw), and isoelectric point (pI) of each UGT protein were calculated by the online program ExPASy (https://web.expasy.org/compute_pi/). Subcellular localization of UGT proteins was predicted using the online analysis tool CELLO v2.5 system (http://cello.life.nctu.edu.tw) from Molecular Bioinformatics Center. The chromosome distribution of *UGT* genes was conducted with the MapChart (v2.32) software.

### Phylogenetic analysis

The ClustalX v2.0 program was used for the alignment of the amino acid sequences of UGTs using the neighbor-joining (NJ) method. A phylogenetic tree was constructed using FigTree v1.4.2 program based on the 145 pomelo UGTs with several functional UGTs, including VvGT5 (GenBank XP_002271025), VvGT6 (NC_012017), VvGT7 (XP_002276546), and VvGT14 (XP_002285770) from grapevine (*Vitis vinifera*); MdPGT1 (XP_008339149) and MdUGT88F1 (EU246349) from apple (*Malus* x *domestica*); SlUGT5 (XP_004231207) from tomato (*Solanum lycopersicum*); F3GT1 (A0A2R6Q8R5) from kiwifruit (*Actinidia chinensis*); AdGT4 (KF954944) from kiwifruit (*Actinidia deliciosa*); FaGT1 (AY663784), FaGT2 (AY663785), FaGT6 (DQ289587), FaGT7 (DQ289588), FaUGT71A33 (XP_004303953.1), FaUGT71A34 (XP_004303954.2), FaUGT71A35 (XP_004303955.1), FaUGT71W2 (XP_011468178.1), FaUGT73B23 (XP 004304022.1), FaUGT73B24 (XP 004304022.1), and FaUGT71K3 (XP 004294260.1) from strawberry (*Fragaria* x *ananassa*); PpUGT85A2 (XP_007227474.1) from peach (*Prunus persica*); GRMZM5G834303 (NP_001148991.2) and GRMZM2G075387 (XP_008670630.1) from maize (*Zea mays*); Cm1,2RhaT (AY048882) from pomelo (*Citrus maxima*); Cs1,6RhaT (DQ119035), CsUGT76F1 (KDO69246.1), CsUGT1 (GQ221686), CsUGT2 (GQ221687), and CsUGT3 (GQ221688) from sweet orange (*Citrus sinensis*). And 19 UGT sequences were from *Arabidopsis thaliana* and obtained from http://www.p450.kvl.dk/UGT.shtml.

### Intron mapping

The pomelo *UGT* intron map was constructed by determining the length, splice site, phase, and position of introns. The exon-intron structure of the *UGT* genes was illustrated with the online Gene Structure Display Server 2.0 program (http://gsds.cbi.pku.edu.cn/) using genomic sequences and CDS sequences. The introns were serially numbered according to their position in the amino acid sequence by aligning all pomelo UGTs. Introns were divided into three types based on their phases: phase 0, phase 1, and phase 2. If the intron positioned between two triplet codons, it was defined as phase 0; If the intron positioned after the first and second bases of a codon, it was defined as phase 1 and phase 2 [20, 32].

### Gene expression analysis

About 0.3 g of flavedo, 0.3 g of albedo, 0.3 g of segment membrane, and 1 g of juice sacs were used for pomelo fruit total RNA extraction according to Zhang et al. [33]. The quality and concentration of RNA samples were determined by NanoDrop One spectrophotometer at A260/A280 absorbance ratio. RNA integrity was verified by gel electrophoresis. Libraries for high-throughput Illumina strand-specific RNA-Seq were prepared as described previously [34]. The data was calculated by reads per kilobase per million mapped read (RPKM) values as transcript abundance. Three biological replicates were performed for gene expression analysis.

### Data analysis

Multi Experiment Viewer (version 4.6.0) was used for heatmap analysis of *UGT* genes transcript abundance and construction of gene clusters in pomelo. VENNY 2.1 online program (https://bioinfogp.cnb.csic.es/tools/venny/index.html) was used to make Venn diagram.

## Supplementary information

**Additional file 1 Table S1.** Information of the pomelo UGTs identified in this study.

**Additional file 2 Table S2.** Predictive information on subcellular localization of pomelo UGTs.

**Additional file 3 Table S3.** Chromosome distribution information of pomelo UGTs.

**Additional file 4 Fig. S1.** Number of pomelo UGTs predicted by subcellular localization.

**Additional file 5 Fig. S2.** Distribution of introns among *UGT* genes in pomelo.

## Data Availability

The raw sequencing reads of transcriptome data in this study are available in the Sequence Read Archive (SRA) database, with the accession number PRJNA663973. All data generated or analysed during this study are included in this published article and its additional files.

## Abbreviations

UGT: UDP-glycosyltransferase
PSPG: Plant secondary product glycosyltransferase
DAB: Days after full blossom
CDS: Coding sequence
RNA-Seq: RNA-Sequencing
