# Supplementary figures and images for: Genome-wide characterization, evolution and expression profiling of UDP-glycosyltransferase family in pomelo (Citrus grandis) fruit
Source: BMC Plant Biol. 2020 Oct 7;20:459. doi: 10.1186/s12870-020-02655-2 (PMC7542425; doi:10.1186/s12870-020-02655-2)

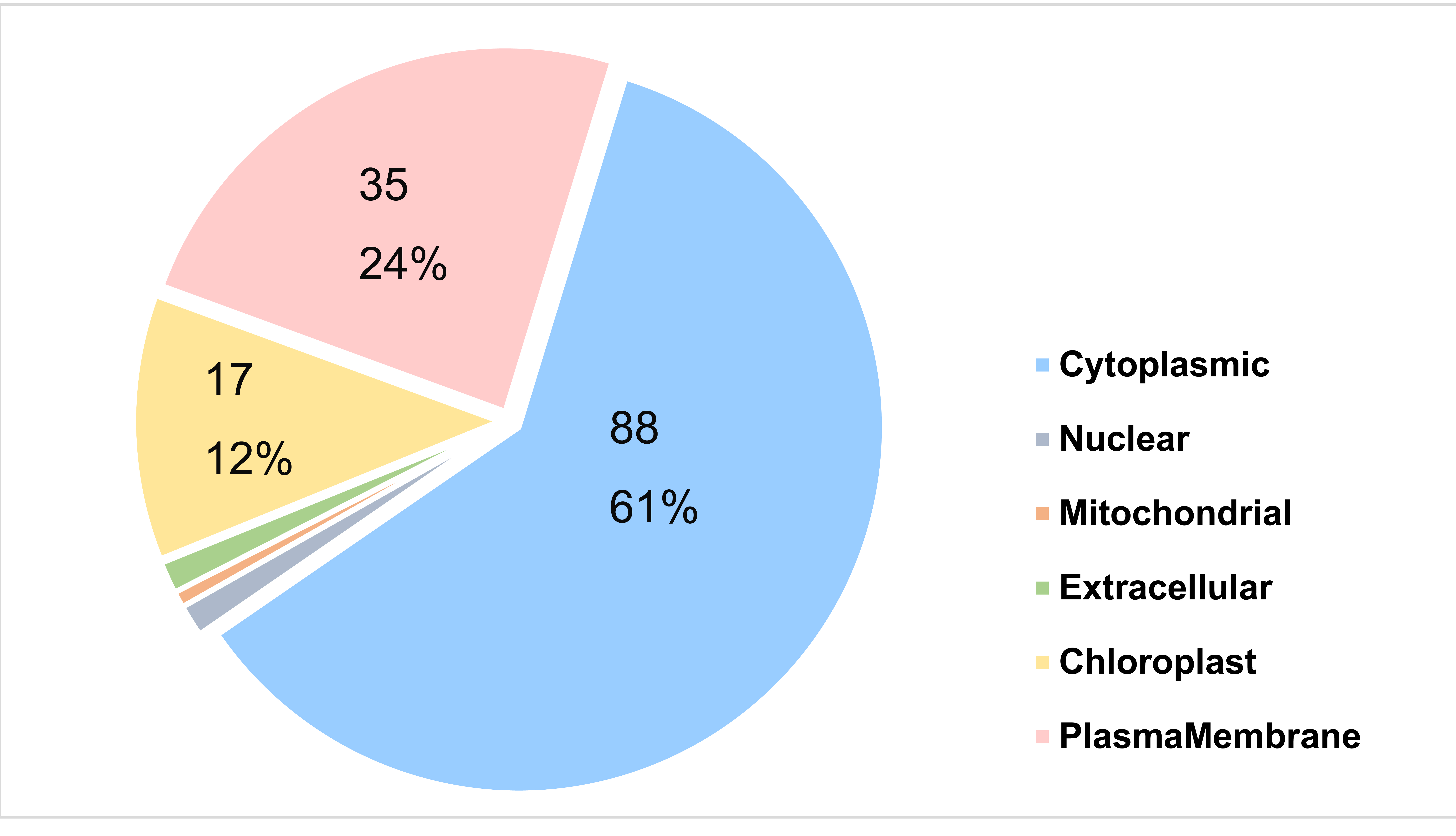

Supplement: Supplementary file 4 — Additional file 4 Fig. S1. Number of pomelo UGTs predicted by subcellular localization. [file 12870_2020_2655_MOESM4_ESM.tif]

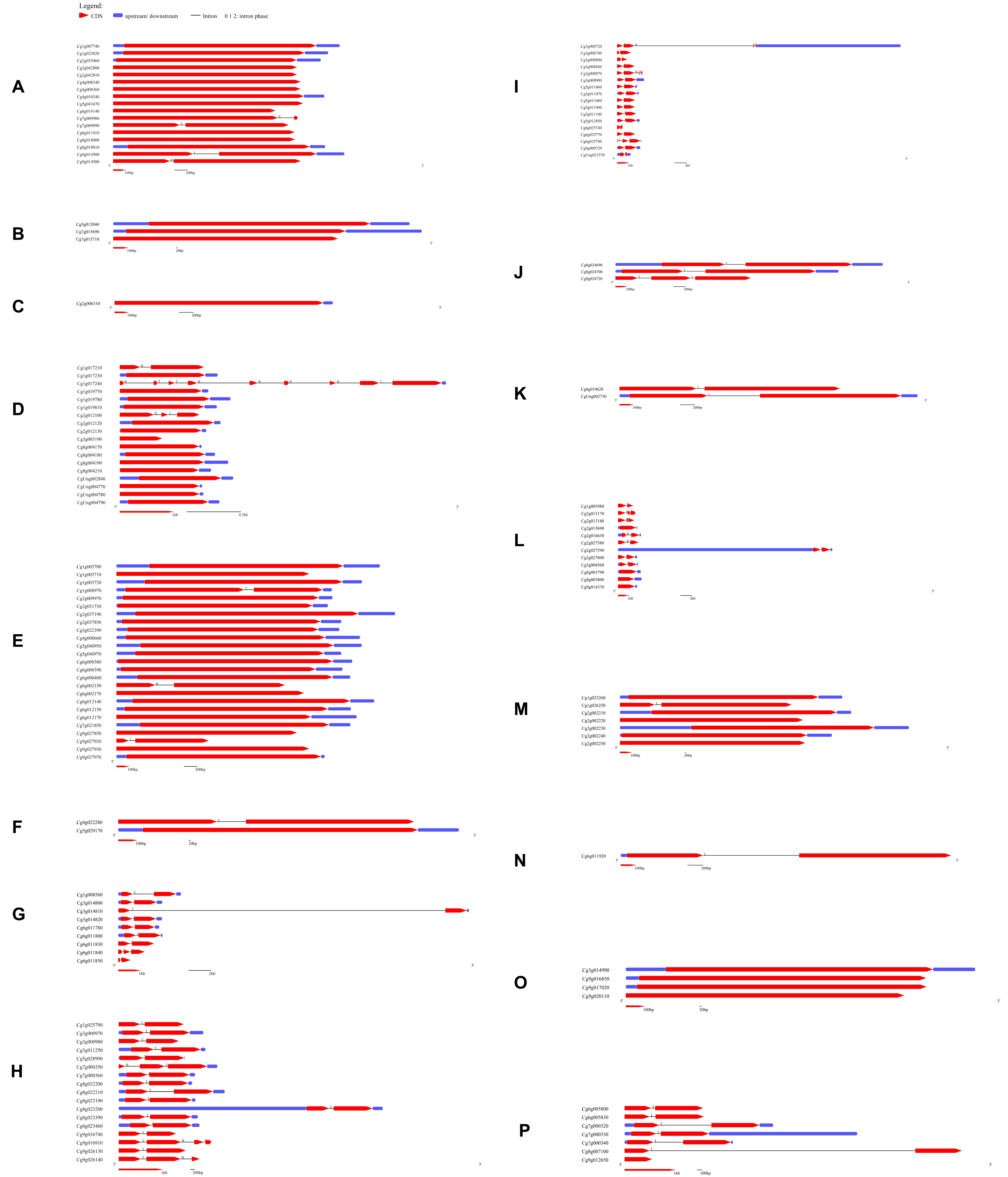

Supplement: Supplementary file 5 — Additional file 5 Fig. S2. Distribution of introns among UGT genes in pomelo. [file 12870_2020_2655_MOESM5_ESM.tif]
